# Role of Neutrophils and Myeloid-Derived Suppressor Cells in Glioma Progression and Treatment Resistance

**DOI:** 10.3390/ijms21061954

**Published:** 2020-03-13

**Authors:** Sabbir Khan, Sandeep Mittal, Kain McGee, Kristin D. Alfaro-Munoz, Nazanin Majd, Veerakumar Balasubramaniyan, John F. de Groot

**Affiliations:** Department of Neuro-Oncology, The University of Texas MD Anderson Cancer Center, 1515 Holcombe Boulevard, Houston, TX 77030, USA; skhan15@mdanderson.org (S.K.); SMittal1@mdanderson.org (S.M.); KMcGee@mdanderson.org (K.M.); KDAlfaro@mdanderson.org (K.D.A.-M.); NKMajd@mdanderson.org (N.M.)

**Keywords:** neutrophils, glioma progression, treatment resistance, myeloid-derived suppressor cells, tumor-associated neutrophils

## Abstract

Recent efforts in brain tumor research have been directed towards the modulation of the immune system for therapeutic interventions. Several human cancers, including gliomas, are infiltrated with immune cell types—including neutrophils and myeloid-derived suppressor cells—that contribute to tumor progression, invasiveness, and treatment resistance. The role of tumor-associated neutrophils and myeloid-derived suppressor cells in cancer biology remains elusive, as these cells can exert a multitude of pro-tumor and antitumor effects. In this review, we provide the current understanding and novel insights on the role of neutrophils and myeloid-derived suppressor cells in glioma progression and treatment resistance, as well as the mechanisms of pleiotropic behaviors in these cells during disease progression, with an emphasis on possible strategies to reprogram these cells towards their antitumor actions.

## 1. Introduction

Gliomas represent approximately 80% of malignant brain tumors, which are classified into four major clinical grades (grades I–IV), on the basis of their histologic characteristics and clinical behavior [1]. In the revised World Health Organization classification of central nervous system tumors, a multidimensional approach was taken for the categorization of gliomas, integrating both histologic and genetic information to define tumor grades and their prognosis [2]. Grade IV glioma or glioblastoma (GBM) is the most common lethal primary brain tumor in adults, with a median survival time ranging from 12 to 15 months, with current standard of care treatment, which includes maximum surgical resection followed by concomitant chemotherapy and radiation therapy (RT). GBM tumors are highly resistant to RT and chemotherapy, and therefore recurrence is inevitable despite an advanced multimodal standard therapy [3]. The current understanding of the complex biology of gliomas is mainly derived from genetic exploration and molecular changes within cancer cells [4,5]. Furthermore, the characterization of the genome, epigenome, and transcriptome of GBM has provided an overall picture of genetic alterations and revealed molecularly distinct GBM subtypes based on gene expression signatures [4,6,7,8,9]. Additionally, single-cell RNA sequencing revealed that multiple subtypes could exist within a tumor, which substantially contributes to the inter- and intra-tumor heterogeneity of GBM/glioma [7].

The glioma microenvironment (GME) is composed of a wide variety of cells, such as differentiated, partially differentiated, and undifferentiated glioma stem cells (GSCs); non-neoplastic stromal cells; endothelial cells; various infiltrating and resident immune cells; and other major cell types of the central nervous system, such as oligodendrocyte progenitor cells, reactive astrocytes, and neurons (reviewed by Hambardzumyan and Bergers [10]). The GME not only harbors various cell types, but also acts as a communication center for the dynamic interaction of tumor and non-tumor cells via direct cell-to-cell contacts or paracrine signaling. Tumor cell and stroma interactions promote tumor growth, immune evasion and therapeutic resistances [11,12].

**Figure 1:**
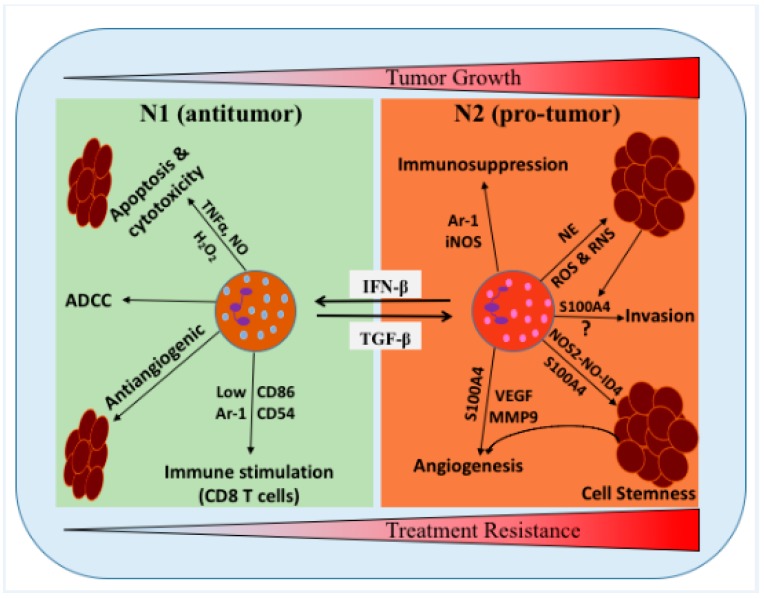
Schematic representation of the proposed roles of neutrophils/tumor-associated neutrophils (TANs) in the glioma microenvironment (GME). Neutrophils can be polarized into two distinct functional phenotypes under certain cytokines and growth factors in the GME, i.e., N1 neutrophils can polarized into N2 in the presence of TGF-β, while N2 neutrophils can polarized into N1 phenotype in the presence of IFN-β. N1 phenotype has been shown to induce tumor cells cytotoxicity/apoptosis, antibody-dependent cellular cytotoxicity (ADCC), activate T cells and inhibit tumor growth. N2 phenotype promoted the tumor growth, stemness, angiogenesis, invasion, and suppress immunity. NE: neutrophil elastase, TNFα: tumor necrosis factor alpha, H_2_O_2_: hydrogen peroxide, MMP9: matrix metallopeptidase 9, NO: nitric oxide, and NOS2: nitric oxide synthase 2.

In the GME, glioma cells secrete several cytokines, chemokines, and growth factors, which attract the infiltration of various myeloid immune cells, differentially activating the microglia, resident immune cells, and endothelial cells. These cells create a specific glioma tumor niche, which promotes tumor growth, invasiveness, and therapy resistance [13,14,15]. Among infiltrated myeloid cells, glioma-associated macrophages, tumor-associated neutrophils (TANs), and myeloid-derived suppressor cells (MDSCs) constitute the major proportion of nonmalignant cells in the GME [16,17,18,19,20]. In glioma, studies have shown that neutrophils have a pro-tumor role, because neutrophilia and an elevated peripheral neutrophil-to-lymphocyte ratio (NLR) are associated with immunosuppression and poor survival and prognosis [18,21,22,23]. Similarly, clinical data from GBM patients have reported infiltration of CD11b^+^/CD14^+^ monocytic and CD11b^+^/CD15^+^ granulocytic subsets of MDSCs in blood and tissue, which is associated with increased glioma grades and poor prognosis [20,24,25]. The antitumor and pro-tumor potential of neutrophils has been reconsidered, owing to the better understanding of their characteristics, such as maturation stage, functional plasticity (N1 vs. N2), and activation stage (Figure 1) [26,27,28,29,30]. In this review, we provide the current understanding and novel insights on the role of neutrophils and MDSCs in glioma progression and treatment resistance, with an emphasis on the possible strategies to reprogram these cells towards their antitumor potential and defer the development of treatment resistance.

## 2. Circulating Neutrophils in Glioma Progression and Treatment Resistance

Neutrophils are the most abundant circulating white blood cells and are the first responders to infection and tissue damage. The primary function of neutrophils is host defense, which occurs through multiple mechanisms, including phagocytosis, release of antimicrobial peptides and proteases, and the formation of neutrophil extracellular traps [31]. Neutrophils are very mobile and are quickly recruited to damaged tissue and invading microbes via proinflammatory signals such as chemokines and lipid mediators, as well as signals to mediate host defense [32,33]. Thus, neutrophils play a critical role in coordinating innate and adaptive immune responses via various mechanisms [34].

Although neutrophils are known to play a crucial role in host pathogen defense and tissue homeostasis, the dysregulation and activation of neutrophils can contribute to chronic inflammation and facilitate tumor growth in various cancers [28,35]. In glioma, increased circulating and tumor-infiltrating neutrophils has been observed in high-grade disease compared with low-grade disease [18,36]. Moreover, immunosuppression in GBM patients is correlated with increased neutrophil degranulation and elevated levels of circulating arginase-1 (Arg1), which is known to have an immunosuppressive effect on T cells [37]. A previous study from our group showed that neutrophils enhanced the proliferation of GSCs via the upregulation of S100A4 expression, which led to tumor growth and resistance against anti-vascular endothelial growth factor (VEGF) therapy in GBM [18]. Neutrophil activation is associated with increased interleukin (IL)-12 (IL-12p70) in GBM patients, which is considered an early sign of tumor progression, and patients with high levels of activated neutrophils had a worse prognosis than those who had low levels [38]. Thus, neutrophil activation has prognostic value for glioma patients and disease outcome.

Bambury et al. reported that pretreatment NLR is associated with overall survival; patients with NLR > 4 had worse overall survival and poorer outcomes than NLR < 4 [22]. Similarly, Mason et al. reported that low NLR was associated with prolonged survival during concurrent treatment with temozolomide (TMZ) and RT, in a cohort of 369 GBM patients [39], independent of other known prognostic factors. Thus, an elevated baseline NLR is associated with poor prognosis in GBM patients [23,39]. Further, NLR is one of the biomarkers of systemic inflammation and considered a poor prognostic factor for many malignancies [23,40]. However, whether this elevated NLR in glioma patients is due to increased neutrophil numbers or decreased lymphocytes population is poorly understood. Since most of the glioma patients have a strong neutrophilia due to overproduction of G-CSF by tumor cells [41,42], an elevated NLR in GBM patients might be due to increased neutrophil count.

In addition, Schernberg et al. reported that pretreatment neutrophilia is negatively associated with overall survival in GBM patients who undergo concurrent treatment with TMZ and RT [43]. Baseline circulatory neutrophil count predicts bevacizumab efficacy in GBM patients, although the role of neutrophils in the antitumor response of anti-VEGF therapy is unclear [44]. A recent study also showed that the absolute baseline number of neutrophils is negatively associated with overall survival, and has a prognostic value for bevacizumab response in patients with recurrent GBM who have not received corticosteroids [45]. This prognostic impact for bevacizumab response is lost in patients who have received corticosteroids during treatment [45].

Taken together, these findings illustrate that increased circulating neutrophils in glioma patients are generally associated with poor overall survival, immunosuppression, promotion of tumor growth, and development of resistance to chemotherapy and RT (Table 1). If prospectively validated, the baseline circulating neutrophil count could be used to predict bevacizumab (anti-VEGF therapy) efficacy in glioma/GBM patients.

## 3. TANs in Glioma Progression

Several human cancers, including GBM, are infiltrated with numerous immune cell types, including neutrophils [42,51]. Interplay between the tumor and immune cells is an emerging key modulator of tumor biology and is a major determinant of pathogenesis and progression of many cancers, including glioma [27,31,42]. Moreover, profound immunosuppression occurs in the tumor microenvironment, particularly in the context of cell-mediated immunity [31], which is driven by an array of cytokines, such as prostaglandin E2, transforming growth factor beta (TGF-β), matrix metallopeptidase 9, IL-10, programmed death-ligand 1 (PD-L1), granulocyte colony stimulating factor, VEGF, and S100A4 [18,31,33,52,53]. At a mechanistic level, most proposed pathways to mediate immunosuppression in GBM are those involving signal transducer and activator of transcription 3 (STAT-3) [54,55], phosphoinositide 3 kinase, Ras–mitogen-activated protein kinase, wingless-related integration site/β-catenin, and indolamine 2, 3-dioxygenase [56].

In addition, tumor microenvironment cytokines recruit TANs and immunosuppressive regulatory T cells to the tumor microenvironment, which results in aggressive tumor growth and development of treatment resistance in many cancers, including glioma [27,57]. Mutant IDH1 glioma tumors, which are less aggressive than wild-type IDH1 tumors, have low TAN infiltration [21]. In particular, neutrophils are recruited at the GBM tumor site by many chemotactic agents, such as IL-8 or chemokine ligand 8 and macrophage migration inhibitory factor [46,58]. Furthermore, TANs also secrete elastase, which can further help in TAN infiltration at the tumor site [59]. TANs promote malignancy in GBM and can mediate angiogenesis via the expression of S1004A within the GME [18]. The depletion of TANs via a monoclonal antibody against Ly6G^+^ has been shown to prolong mouse survival in a preclinical mouse model of GBM [47]. Interestingly, depleting the Ly6G^+^ also reduced the number of CD4^+^-FoxP3^+^ cells in the tumor site, suggesting an alteration of tumor microenvironment upon depletion of LY6G^+^ cells. Taken together, this suggests that TANs played a significant role in the promotion of gliomagenesis in the de novo gliomas murine model [47]. However, the complete mechanism of TAN recruitment and the role of TANs in tumor growth are still not completely understood in GBM. How the heterogeneity of the tumor microenvironment affects neutrophil reprogramming and/or tumor cells following their extravasation into the tumor niche still needs to be unraveled.

## 4. TANs and Treatment Resistance in Glioma

Despite a better understanding of tumor heterogeneity and molecular pathology of glioma at the transcriptomic level, there have been limited advances in the treatment of glioma over the past three decades. Three critical factors hamper successful treatment of glioma and lead to the development of treatment resistance: GSCs, tumor heterogeneity, and microenvironmental niches, including various infiltrating immune cells. In this regard, bidirectional signaling of TANs and the tumor microenvironment can reprogram the functional plasticity of TANs and modulate tumor heterogeneity, which can affect glioma treatment outcomes (Table 1). Current understanding of the involvement of TANs in the development of treatment resistance against the various targeted therapies for glioma is discussed in the following sections.

### 4.1. Chemotherapy and Anti-VEGF Therapy Resistance

The current treatment for malignant glioma is surgical resection in combination with RT and chemotherapy, which includes TMZ and nitrosoureas alkylating agents [60,61]. There is clear evidence of significant infiltration of TANs in wild-type IDH1 tumors, and this is correlated with poor prognosis and decreased overall survival in GBM patients [21,36,62]. However, no clear functional mechanistic studies have established the role of TANs in standard chemotherapy (TMZ and nitrosoureas) treatment resistance. As mentioned above, increased circulating neutrophils (NLR) are associated with poor outcome of treatment with TMZ in glioma patients (see Section 2). Moreover, in other cancer types, a growing body of evidence has demonstrated that TANs are associated with pro-tumor phenotypes and play a role in the development of chemotherapy resistance [27,28,63]. Thus, future experimental studies are warranted to investigate the role of TANs in the development of resistance against standard chemotherapy (TMZ) in GBM.

Bevacizumab is associated with improved progression free survival and is commonly used in GBM patients for reduction of cerebral edema and symptom control [64]. However, resistance to anti-VEGF therapy has been shown to be associated with myeloid cell infiltration and mesenchymal transition in GBM [65,66]. Recently, we have also reported that increased neutrophil infiltration promotes tumor progression and resistance to anti-VEGF therapy in GBM [18]. Therefore, it is possible that TANs or their derived substances can modulate the tumor microenvironment to develop resistance against anti-VEGF therapy. However, further studies are needed to determine the exact mechanisms of TAN recruitment to mediate the development of resistance to anti-VEGF therapy in GBM.

### 4.2. RT Resistance

RT is the cornerstone of treatment for both low-grade and high-grade gliomas. As mentioned above, increased circulating neutrophils (NLR) have been associated with poor survival and prognostic outcomes in patients who received RT [39]; therefore, TANs may be involved in the development of resistance against RT and diseases recurrence. Recently, it has been reported that radiation-induced senescence in GBM cells promotes the recruitment of Ly6G^+^ (TANs) and modulates tumor microenvironmental cells and vessel formation through nuclear factor kappa-light-chain-enhancer of activated B cell (NFκB) signaling [49,67]. Palumbo et al. showed that infiltrating Ly6G^+^ cells support the conversion of GBM tumor cells to a more stem-like state through dedifferentiation and nitrosative stress (NOS2-NO-ID4) signaling [48]. Jeon et al. further confirmed this mechanism by showing that NFκB inhibitors and Ly6G-neutralizing antibodies reduced the number of GSCs and prolonged the survival of tumor-bearing mice after RT [49]. Furthermore, Ly6G^+^ cells positively correlated with NOS2-NO-ID4 signaling in newly diagnosed and recurrent GBM patients [49]. However, direct clinical evidence establishing a clear role of TANs in the development of resistance to RT is still lacking. Moreover, it is well established that GBM tumor cells with stemness properties are resistant to RT [48,68,69,70,71,72]. Infiltrating TANs secrete S100A4 and promote the growth of GSCs and a malignant phenotype, as well as drug resistance, in in vitro and mouse models of GBM [18]. Therefore, TANs might contribute to the development of resistance to RT via S100A4-mediated increased cell proliferation and stemness in GSCs. Future studies are needed to explore the possible mechanistic role of S100A4 in the development of resistance to RT (Table 1).

### 4.3. Immunotherapy Resistance

Immunotherapy is quite successful for the treatment of many solid cancers [73]. In glioma, several randomized clinical trials have demonstrated limited success for immunotherapies, such as targeted vaccines and checkpoint inhibitors; many challenges must be overcome for successful clinical translation of these therapies in GBM [57,74,75,76]. The unique immunosuppressive tumor microenvironment of the central nervous system is at least in part the reason for limited success of immunotherapeutics for gliomas [42,75,76]. TANs are recruited at the tumor site by chemotactic agents (chemokine legend 8, macrophage migration inhibitory factor, and osteopontin) produced by tumor cells and/or other non-tumor cells present in the microenvironment, and this partly contributes to the immunosuppressive environment within the tumor [42,50,62,77]. Moreover, a comparative analysis of immune cell fractions among the molecular subtypes of GBM using CIBERSORT indicated that mesenchymal tumors have a significantly higher number of TANs than do other subtypes [50]. Thus, increased TANs in mesenchymal tumors might contribute to the extreme immunosuppressive environment and plausibly mediate immunotherapy resistance. In contrast, Chang et al. reported that virus-stimulated neutrophils in the tumor microenvironment increase T cell-mediated antitumor immunity in a mouse model of melanoma [78]. Although TANs are associated with an immunosuppressive environment within the tumor, clear experimental and clinical evidence is lacking to establish the involvement of TANs in immunotherapy outcomes, which is likely context dependent.

## 5. MDSCs in Glioma Progression

MDSCs are important cells in the tumor immune microenvironment and are correlated with cancer stage, metastasis, and therapeutic resistance [79]. However, such information in glioma is very limited [20,24]. In 2007, Gr-1^+^ Cd11b immune infiltrates were renamed as MDSC, owing to their ability to suppress the activation and function of T cells [80,81]. MDSCs encompass early myeloid progenitors that have a granulocytic or monocytic lineage [82]. During myelopoiesis, hematopoietic stem cells differentiate into common myeloid precursor cells and immature myeloid cells. Under normal conditions, immature myeloid cells differentiate into mature myeloid cells, such as macrophages and dendritic cells. However, in pathologic conditions such as sepsis, trauma, autoimmune diseases, infectious diseases, and cancer, immature myeloid cells do not differentiate into mature cells and can form MDSCs, depending on the pathology-specific cytokines [83,84].

Despite their abundance in various pathologies, the characterization of MDSCs remains mainly descriptive owing to a lack of unique markers. Recently, efforts have been made to improve the nomenclature and characterization standards of MDSCs and to create a harmonized staining and gating procedure for the analysis of human MDSCs [85,86]. Basically, MDSCs have been divided into three major subgroups, based on surface markers: monocytic MDSCs, granulocytic polymorphonuclear MDSCs (PMN-MDSCs), and early immature MDSCs [82,85]. In mice, these MDSCs can be characterized by CD11b^+^Ly6C^high^Ly6G^−^ as monocytic MDSCs and CD11b^+^Ly6C^low^Ly6G^+^ as PMN-MDSCs [87]. In contrast to mice, humans do not express Ly6G and their MDSCs have been characterized by the presence of CD14 on monocytic MDSCs and CD15, along with CD66b on PMN-MDSCs. All three types of MDSCs express CD33 along with these markers; thus monocytic MDSCs can be further distinguished by the selective expression of CD14 and HLA-DR [81,85,88].

MDSCs have been shown to expand systemically in various pathologies such as infection [89,90], sepsis [91], and autoimmune disorders [92]. All the major cytokines that are involved in the normal hematopoiesis and proliferation of MDSCs, such as macrophage colony stimulating factor and granulocyte macrophage colony stimulating factor (GM-CSF), are highly expressed in GBM [93]. In GBM patient blood, studies have shown high levels of neutrophilic and monocytic subsets of circulating MDSCs in the peripheral blood, compared with that of healthy donors [20,24,94]. A comparison between GBM patients and age-matched healthy donors and other cancer patients revealed that CD15^+^CD14^−^ PMN-MDSCs were significantly increased in GBM patients compared with the heathy controls [20]. Similarly, in GBM tumor masses, the predominant presence of neutrophilic (CD33^+^CD15^+^CD14^−^HLA-DR^−^) and negative lineage (CD33^+^CD15^−^CD14^−^HLA-DR^−^) MDSCs has been shown [17,24]. However, MDSCs from both patients and healthy donors had a similar expression pattern of myeloid markers, such as CD124, CD86, and CD40, with the absence of myeloid activation markers viz B7-1/CD80 and PD-L1 [17,24]. Recently, a multicolor flow cytometry-based analysis of peripheral blood and tissue of 52 GBM patients revealed an increased frequency of monocytic MDSCs (CD14^high^CD15^+^) and PMN-MDSCs (CD14^low^CD15^+^) in the blood and tumor masses [95]. Furthermore, a comprehensive analysis of the blood cells of these patients revealed that the cells which expressed CD14^low^CD15^high^ (PMN-MDSCs) suppressed T cell function, while the cells with CD16^low^HLA-DR^high^ MDSCs did not inhibit T cell proliferation in vitro [95]. Furthermore, there was a strong correlation between the number of PMN-MDSCs and CD4^+^ effector memory T cells in GBM tumors. The CD4^+^ T cells were functionally exhausted and found to express high levels of PD-1 with concomitant increased levels of PD-L1 in tumor-derived MDSCs [95].

In a mouse model of glioma, a monocytic population of MDSCs (CD11b^+^Gr1^low^) has been found to infiltrate more than the granulocytic (CD11b^+^Gr1^high^) MDSCs, which could simultaneously express the marker of pro-inflammatory M1 and tumor-supportive M2 macrophages [17,96,97,98]. These populations constituted about 8% of total tumor cells and also expressed CD11c and IL-4Rα. The depletion of these cells was found to improve the survival of mice, suggesting that these cells had a tumor-promoting role in GBM [17,98]. All of the above studies indicate that different MDSC subsets can accumulate in the blood and tissue of GBM patients, and these subsets of MDSCs can play a significant role in shaping the GBM tumor microenvironment, as well as promoting immunosuppression in GBM.

Several tumor-associated factors and growth factors have been shown to affect the recruitment of MDSCs from the peripheral blood and the promotion of their immunostimulatory phenotype to exert their immunosuppressive effects [99,100]. The proliferation and migration of MDSCs is controlled by the activation of STAT-3, which regulates the expression of S100A8 and S100A9 [101]. Furthermore, various studies have shown that MDSCs require various recruitment factors such as C-C motif chemokine ligand 2, VEGF receptor 2, IL-8, and galectin-1 and activation factors such as GM-CSF, stimulator of interferon (IFN) genes, CD40, and IL-12, to exert immunosuppression at full potential. In addition, some other factors, such as macrophage colony stimulating factor, phosphoinositide 3 kinase, receptor tyrosine kinases, and cyclooxygenase, have also been shown to promote the formation of MDSCs from myeloid precursor cells [99,100].

## 6. MDSCs and Treatment Resistance in Glioma

In recent years, MDSCs have emerged in the cancer field as a powerful modulator of the immune system; MDSCs control not only T cell activity but also recovery from an immunologic insult [102]. Accumulation of MDSCs has been linked to increasing grades of glioma. Thus, understanding the role of MDSCs in therapeutic resistance in glioma could pave a path to establish novel targets for the treatment of GBM.

### 6.1. MDSC-Mediated Immunosuppression and Therapy Resistance in Glioma

Myeloid cells are an important part of innate immunity and play a significant role in immunotherapy. However, in pathologic conditions such as cancer, myeloid cells become immunosuppressive, owing to the presence of various cytokines/chemokines. These immunosuppressive cells inhibit the activity of antitumor immune cells and lead to immunotherapy resistance in cancer patients [93]. Various peripheral immune cells such as glioma-associated myeloid cells and macrophages, MDSCs, and regulatory T cells have been shown to inhibit the host’s antitumor response in GBM patients [15].

Although much information is available regarding immunosuppression mediated by glioma-associated myeloid cells and macrophages in GBM, for MDSCs, the detailed mechanism is still being unraveled [13,103]. In other solid tumors, in general, MDSCs exert immunosuppression through the inhibition of T cell activity [93]. However, the interaction of MDSCs with other antitumor immune cells, such as natural killer (NK) cells, dendritic cells, and pro-inflammatory macrophages has also been reported [17,20,58,104,105,106]. MDSC-mediated T cell suppression is mainly exerted through the depletion of either L-arginine (important for T cell proliferation) or cysteine (required for T cell activation). Arg1 is an essential enzyme that metabolizes L-arginine into L-ornithine in the urea cycle [107,108]. In the tumor microenvironment, MDSCs generate increased amounts of reactive oxygen species (ROS) and reactive nitrogen species, which subsequently upregulate the expression of Arg1 and inducible nitric oxide synthase (iNOS), respectively. Arg1 and iNOS at increased levels metabolize the available L-arginine in the tumor microenvironment, leading to the depletion of L-arginine and diminished T cell-mediated antitumor activity. Specifically, ROS-mediated depletion of L-arginine leads to the cell cycle arrest of T cells and the downregulation of CD3 ζ-chain expression. The reduction of CD3 ζ makes the T cells anergic, through nitrosylation of T cell receptors [99]. In contrast, reactive nitrogen species lead to increased nitric oxide and subsequently nitrosylation of IL-2 pathway mediators, leading to T cell suppression. Furthermore, increased levels of ROS have also been shown to induce T cell apoptosis. Reactive nitrogen species such as peroxynitrite can nitrosylate C-C motif chemokine ligand 2 present in the tumor microenvironment. This modified C-C motif chemokine ligand 2 can attract MDSCs but not CD4+ and CD8+, which can explain the selective accumulation of MDSCs in the tumor area [100]. Furthermore, increased production of hypoxia-inducible factor 1α in the hypoxic environment of the tumor can induce the expression of PDL-1 on MDSCs, which can further inhibit T cell activity through PD-1/PD-L1 interaction [101].

ROS production in cells could be mediated through several mechanisms, but in MDSCs, studies have shown that STAT-3 plays a major role in the production of increased ROS through increased NADPH oxidase (NOS) 2 activity. Furthermore, L-arginine depletion by Arg1 triggers the production of ROS through iNOS. Several tumor-derived growth factors, such as TGF-β, IL-6, IL-10, and GM-CSF, can increase the production of ROS [92].

Cysteine is an essential component required for T cell activation. However, T cells cannot synthesize cysteine; instead, it is delivered by antigen-presenting dendritic cells and macrophages which convert methionine and cystine to cysteine [109]. Similarly, MDSCs can also import cystine, but they cannot deliver the cysteine, which leads to a shortage of cystine in dendritic cells and macrophages and ultimately cysteine deprivation in T cells [105]. MDSCs can also induce the conversion of naïve CD4+ T cells into regulatory T cells through CD40–CD40L interactions; production of IFNγ, IL-10, and TGF-β; and Arg1 expression. Furthermore, by producing TGF-β1 and retinoic acid, MDSCs can also promote the trans-differentiation of Th17 into forkhead box P3 regulatory T cells [104,109]. MDSC-mediated dendritic cell impairment requires the production of IL-10, which inhibits dendritic cell maturation and concomitantly IL-12 production [110].

Owing to increased inflammation in the tumor, MDSCs have the ability to skew the macrophage phenotype from type 1 to type 2 through the IL-12-dependent toll-like receptor 4 pathway. Type 2 macrophages create an immunosuppressive environment, thus promoting the progression of cancer cells [110,111,112]. MDSCs can also regulate NK cell function through MDSC interaction, with natural cytotoxicity triggering receptor 3 (NRC3/NKp30) and NK group 2D receptors on NK cells in a cell contact-dependent manner [113,114]. MDSCs downregulate the expression of NKp30 and NK group 2D on NK cells through TGF-β1 present on the membrane, which leads to decreased IFNγ production and ultimately reduced NK cell activity.

### 6.2. MDSCs and RT Resistance

RT has been shown to prolong the life of glioma patients [115]. RT alters the tumor microenvironement by increasing infiltration of immunosuppressive myeloid cells and release of tumor antigens from necrotic tissue [116,117]. It is crucial to understand the interplay between RT and the tumor microenvironement to integrate immunotherapeutic approaches into the current standard of care for gliomas.

The irradiation of human and murine cells has been shown to upregulate the expression of various immune molecules, such as Fas, intercellular adhesion molecule 1, MHC class I, carcinoma-associated antigens, and mucin-1, to target them for immune phagocytic cells [118,119,120,121]. In 2006, Newcomb et al. showed that RT can be used to enhance the expression of MHC class I in GL261 cells that were retrovirally transduced to release GM-CSF. This combination treatment primed myeloid cells towards an immunostimulatory state and rendered the glioma more sensitive to RT compared with RT alone [122].

Nanomaterials have been used as radio-sensitizers for cancer treatment, with minimal side effects [123,124,125]. Metal-based nanomaterials can generate photoelectrons to enhance the dose effect of RT and induce cytotoxicity in tumor cells through increased ROS production [123,124,125]. In a recent study, Wu et al. showed that the combination of RT and a magnetic nanoparticle-based platform with cationic polymer modification induced cytotoxicity in glioma cells and increased the median survival of immunocompetent and athymic glioma mice [126]. This observed effect was attributed to the repolarization of MDSCs towards the pro-inflammatory phenotype from the immunosuppressive phenotype and to the increased expression of tumor necrosis factor-α and iNOS. In addition, MDSCs taken up by the nanoparticles could then be delivered to the tumor microenvironment to enhance the production of ROS to increase the efficacy of RT. RT can be used to modulate the accumulation and distribution of nanoparticles to enhance drug delivery and antitumor efficacy [127]. Apart from X-rays, other types of radiation, such as carbon irradiation and proton irradiation, have also evolved, with the aim of increasing the target dose in cancer tissue while minimizing collateral damage to normal tissue [128,129].

## 7. Conclusions and Future Perspectives

It has become increasingly clear that TANs play a major role in cancer biology and treatment. Despite the large amount of literature suggesting that neutrophils have pro-tumor effects, evidence from multiple studies has also shown that these cells can be reprogrammed to kill tumor cells [28,30,33,52,63,130]. However, in glioma, increased circulating neutrophils and TANs have been observed, particularly in high-grade disease, and this is associated with poor overall survival, immunosuppression, promotion of tumor growth, and development of resistance to chemo-radiotherapy and bevacizumab (anti-VEGF therapy).

Although neutrophils are associated with immunosuppression, their involvement in immunotherapy outcomes is unknown. TANs are known to exhibit both pro-tumor (N2-like) and antitumor (N1-like) functional phenotypes within the tumor in lung cancers [130] and mesothelioma [29]. However, the characterization of functional plasticity (N1 vs. N2) of TANs in glioma/GBM is lacking. Therefore, it will be important to study the molecular mechanisms that may promote the differentiation of TANs into an antitumor phenotype (N1), such as stimulation of type I IFN and/or inhibition of TGF-β signaling (Figure 1) [29].

MDSCs have been used as prognostic markers in various other cancers, such as melanoma, kidney cancer, prostate cancer, and breast cancer, but less information is available about their role in glioma [131,132]. Studies have shown that the presence of CD15+ MDSCs, along with M2 macrophages, can be used as a marker of glioma grade [133]. Furthermore, increased circulating MDSCs have been associated with poor prognosis and survival in GBM patients [134]. However, mechanistic and functional studies of the interaction between MDSCs and glioma, along with immunosuppression of antitumor cells at the GBM site, are still lacking.

Several clinical trials employing myeloid cell-related factors in combination with other therapies are currently ongoing for several cancers, and preliminary results from some of these clinical trials have shown that this approach has promising immunologic efficacy [100]. On the basis of these trials, clinical trials of combination treatments with GM-CSF with EGFRvIII peptide vaccine and bevacizumab have shown that this combination improved progression-free survival in GBM patients compared with single-modality treatment [135]. Similarly, the preliminary results of a phase II clinical trial of combination treatment with GM-CSF with cyclophosphamide and bevacizumab, as well as results from a phase I trial of combination treatment with MK-1454 (stimulator of IFN gene agonist) and pembrolizumab, and results from a completed trial of Toca 511 and Toca FC, have shown that these treatments increased survival rates in GBM patients [136,137].

Based on the current pre-clinical and clinical studies, targeting MDSCs could be integrated with standard or immunotherapies as complementary treatment strategies for the effective treatment of glioma. However, additional research into the immunosuppressive and pro-tumoral role of MDSCs in glioma is needed.

## Figures and Tables

**Table 1:** Experimental and clinical evidence, highlighting the role of circulating neutrophils and tumor-associated neutrophils (TANs) in glioma cell proliferation and growth, stemness of glioma stem cells (GSCs), angiogenesis, and therapeutic resistance.

Mechanism/Inference	Test Systems	Specific Cells Used	References
Glioma-derived factors affect circulating neutrophils and influence their infiltration into the tumors	In vivo human	Blood neutrophils and tumor sections	[36]
Neutrophils enhance proliferation of GSCs and promote glioma progression and resistance against anti-vascular endothelial growth factor (VEGF) therapy via upregulation of S100A4	Mixed (in vitro and in vivo in both human and mouse)	Tumor tissue microarray, GSCs and mouse xenografts	[18]
Neutrophil degranulation is associated with elevated levels of circulating Arg1, which promotes tumor growth and immunosuppression	In vitro and in vivo human	Blood neutrophils and tumor sections	[37]
Increased neutrophil activation levels indicate early signs of tumor progression and provide prognostic value in glioblastoma (GBM)	In vivo human	Blood neutrophils and serum	[38]
Immunosuppression within the tumor is driven by the overexpression and production of G-CSF and S100A4	Mixed (in vitro and in vivo in both human and mouse)	Glioma cells, GSCs and blood samples	[18,41]
IL-6 and IL-8 partially mediated by glioma cells have a protective effect on blood neutrophils	In vitro human	Blood neutrophils and glioma cells	[46]
Depletion of neutrophils via monoclonal antibody against Ly6G prolongs the survival of mice with developing gliomas	Mixed (in vitro and in vivo in mouse, and in vitro human)	Transgenic mice and patients’ blood	[47]
TANs are associated with tumor aggressiveness in mutant-IDH1 glioma	Mixed (in vivo mouse and human)	Transgenic mice, patients tumor tissue and blood cells/RNA	[21]
Primary glioma cells sustaining NOS2 activity promote proliferation, migration, and neurosphere generation and represent a prognostic factor for glioma malignancy and recurrence	Human in vitro	Glioma cell lines and primary culture	[48]
Radiation-induced infiltrating Ly6G+ neutrophils support the conversion of GBM tumor cells to GSCs via the regulation of nitrosative stress and dedifferentiation (NOS2-NO-ID4) signaling in newly diagnosed/recurrent GBM patients, and this is negatively associated with survival and radiation therapy outcomes	Mixed (in vitro and in vivo in both human and mouse)	Human glioma cell lines, tumor single cells, and glioma mouse models	[49]
In a CIBERSORT comparative analysis of immune cell fractions, mesenchymal subtypes of GBM have higher levels of TANs than other subtypes	Human in vitro and in vivo	GSCs and GBM tumor tissue	[50]

Arg1: arginase-1, GBM: glioblastoma, G-CSF: granulocyte colony stimulating factor, IL: interleukin, NOS2: nitric oxide synthase 2, PMNs: polymorphonuclear leukocytes, VEGF: vascular endothelial growth factor. Term ‘mixed’ indicated utilization of both human and mouse cells/tissue in the experiments.

## Abbreviations

Arg1	Arginase-1
GBM	Glioblastoma
GM-CSF	Granulocyte macrophage colony stimulating factor
GME	Glioma microenvironment
GSCs	Glioma stem cells
IFN	Interferon
IL	Interleukin
iNOS	Inducible nitric oxide synthase
MDSCs	Myeloid-derived suppressor cells
NFκB	Nuclear factor kappa-light-chain-enhancer of activated B cells
NK cells	Natural killer cells
NLR	Neutrophil-to-lymphocyte ratio
PD-L1	Programmed death-ligand 1
PMN-MDSCs	Granulocytic polymorphonuclear MDSCs
ROS	Reactive oxygen species
RT	Radiation therapy
STAT-3	Signal transducer and activator of transcription 3
TANs	Tumor-associated neutrophils
TGF-β	Transforming growth factor beta
TMZ	Temozolomide
VEGF	Vascular endothelial growth factor
